# Supplementary material for: Distinct spatially organized striatum-wide acetylcholine dynamics for the learning and extinction of Pavlovian associations
Source: Nat Commun. 2025 Jun 4;16:5169. doi: 10.1038/s41467-025-60462-5 (PMC12137636; doi:10.1038/s41467-025-60462-5)
Supplement: Supplementary file 1 — Supplementary Information [file 41467_2025_60462_MOESM1_ESM.pdf]

Distinct spatially organized striatum-wide acetylcholine dynamics for the learning and extinction of Pavlovian associations

Bouabid et al - Supplementary information

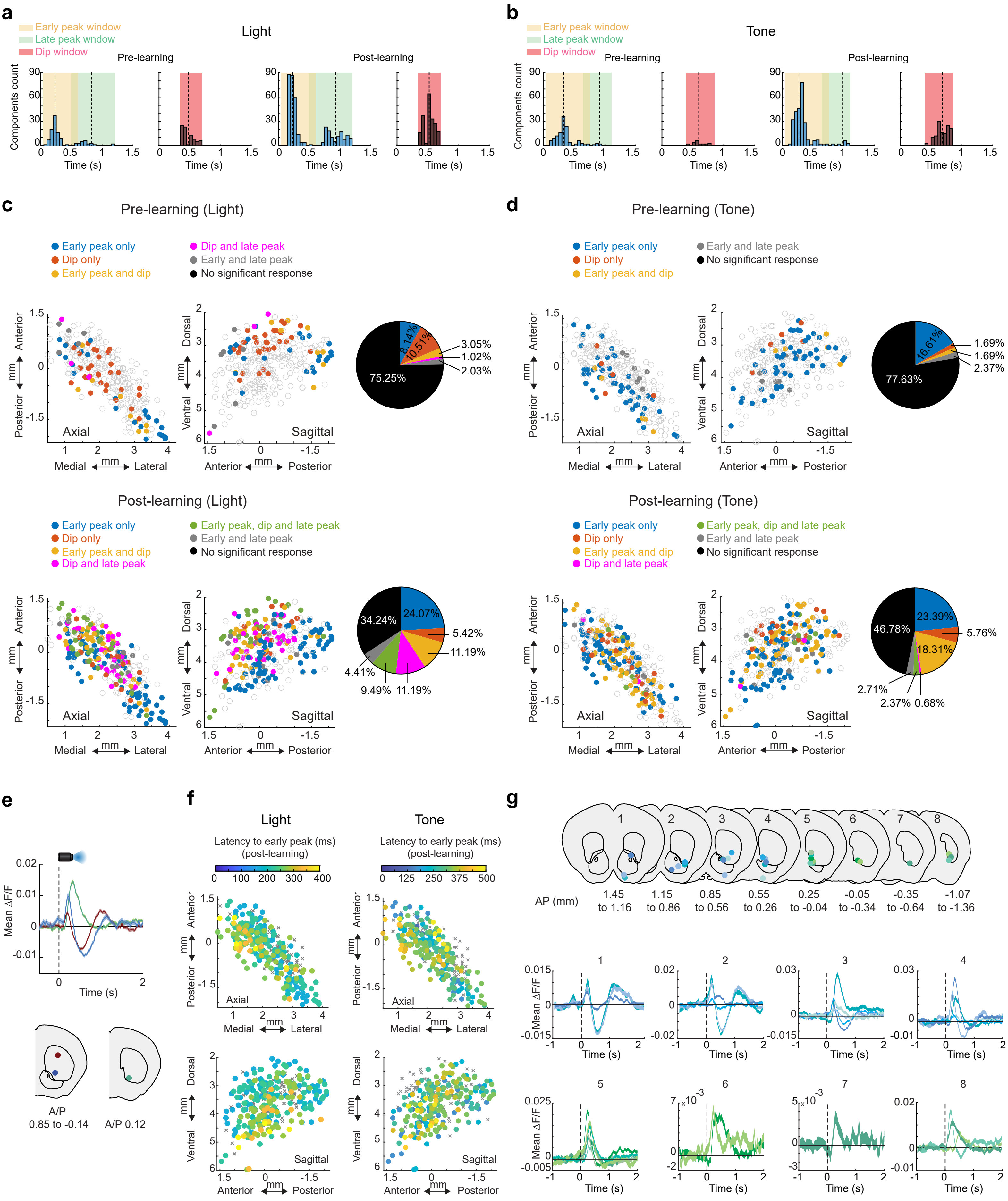

a
Light
Early peak window
Late peak wndow
Dip window
Pre-learning
Post-learning
Components count
Time (s)
b
Tone
c
Pre-learning (Light)
Early peak only
Dip only
Early peak and dip
Dip and late peak
Early and late peak
No significant response
Axial
Sagittal
Anterior
Posterior
Dorsal
Ventral
Medial
Lateral
mm
75.25%
8.14%
10.51%
3.05%
1.02%
2.03%
Post-learning (Light)
Early peak, dip and late peak
34.24%
24.07%
5.42%
11.19%
4.41%
9.49%
11.19%
d
Pre-learning (Tone)
77.63%
16.61%
1.69%
1.69%
2.37%
Post-learning (Tone)
46.78%
23.39%
5.76%
18.31%
2.71%
2.37%
0.68%
e
Mean ΔF/F
Time (s)
A/P
0.85 to -0.14
A/P 0.12
f
Light
Tone
Latency to early peak (ms)
(post-learning)
g
AP (mm)
1.45 to 1.16
1.15 to 0.86
0.85 to 0.56
0.55 to 0.26
0.25 to -0.04
-0.05 to -0.34
-0.35 to -0.64
-1.07 to -1.36

Supplementary Fig. 1: Additional quantification of component specific changes in cue-evoked ACh signals and mutant ACh-sensor controls during Pavlovian learning. a Histogram of latencies to significant (see Methods) peaks and troughs to light cue onset pre (left) and post (right) learning. Shaded regions indicate time windows used to identify each component (early peak, dip, and late peak), and the dashed lines indicate the means (pre learning: early peak 0.23± 0.008s, dip 0.47±0.009s, late peak 0.82±0.045s; post learning: early peak 0.23±0.003s, dip 0.52±0.005s, late peak 0.93±0.012s). **b** Same as (**a**) for the tone cue (pre learning: early peak 0.32±0.01s, dip 0.6±0.02s, late peak 0.91±0.02s; post learning: early peak 0.23±0.005s, dip 0.52±0.009s, late peak 0.93±0.03s). Two-tailed t-test for light vs. tone component latencies: pre learning early peak: p= 9.3×10^{-10}, post learning early peak p = 3.78×10^{-19}; pre learning dip p = 2.07×10^{-7}, post learning dip p = 1.97×10^{-41}; pre learning late peak p = 0.47, post learning late peak p = 0.33. **c** Maps (axial left, sagittal right) indicating the presence of each combination of signal components to light cue onset for each fiber (circle) pre (top) and post (bottom) learning. Empty circles are fibers with no significant response. Pie charts indicate percentages of fibers with each combination of signal components for light cue onset pre (top) post (bottom) learning. **d** Same as (**c**) for the tone cue. **e** Mean light cue triggered ∆F/F for post learning trials for three representative fibers in the same mouse (locations in coronal sections at bottom) illustrating the larger magnitude and longer latency ACh peaks in more ventral and posterior locations and relatively larger dips in more anterior and dorsal regions, consistent with the patterns shown in Fig. 2. **f** Maps (axial top, sagittal bottom) of latencies to early peaks to light (left) and tone (right) cue onsets post learning for all fibers. Xs are fibers with no significant early peak. **g** Light cue onset-triggered ∆F/F averages (bottom) for 29 ventral striatum fibers in 8 mice on post-learning trials (n = 40-100 trials). Colors of the traces correspond to the fiber locations shown in the coronal plane schematics on top, where blue colors correspond to more anterior locations and green colors to more posterior locations. Shaded regions, S.E.M. Brain schematics in (e) and (g) were adapted from the Allen Mouse Brain Common Coordinate Framework (CCFv3) https://atlas.brain-map.org/. Source data are provided as a Source data file.

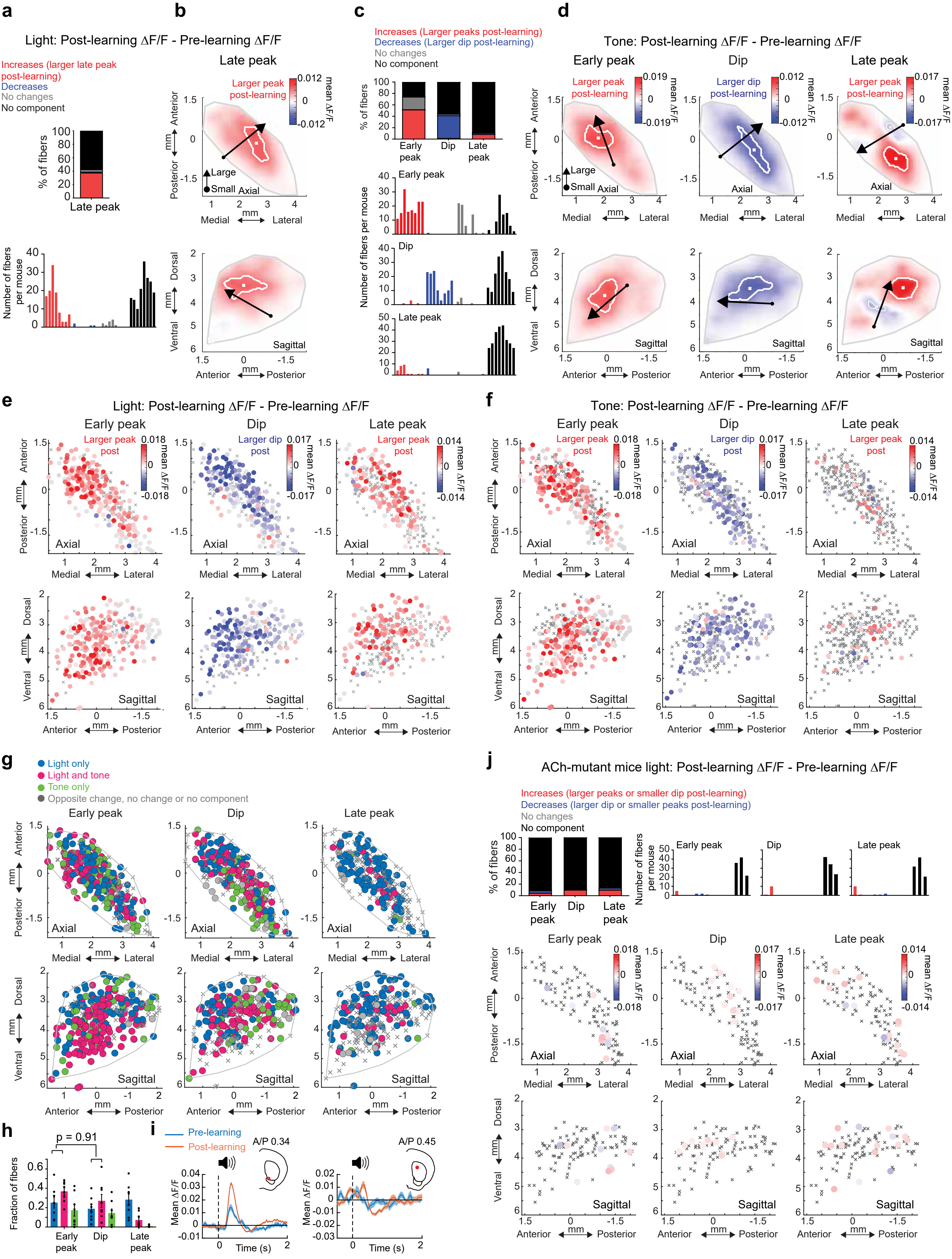
a
Light: Post-learning ΔF/F - Pre-learning ΔF/F
Increases (larger late peak post-learning)
Decreases
No changes
No component
% of fibers
Late peak
Number of fibers per mouse
b
Late peak
Larger peak post-learning
mean ΔF/F
0.012
-0.012
Anterior
Posterior
Large
Small
Axial
Medial
Lateral
mm
Dorsal
Ventral
Sagittal
Anterior
Posterior
c
Increases (Larger peaks post-learning)
Decreases (Larger dip post-learning)
No changes
No component
% of fibers
Early peak
Dip
Late peak
Number of fibers per mouse
d
Tone: Post-learning ΔF/F - Pre-learning ΔF/F
Early peak
Dip
Late peak
Larger peak post-learning
Larger dip post-learning
0.019
-0.019
0.012
-0.012
0.017
-0.017
e
Light: Post-learning ΔF/F - Pre-learning ΔF/F
Early peak
Dip
Late peak
Larger peak post
Larger dip post
0.018
-0.018
0.017
-0.017
0.014
-0.014
f
Tone: Post-learning ΔF/F - Pre-learning ΔF/F
g
Light only
Light and tone
Tone only
Opposite change, no change or no component
h
p = 0.91
Fraction of fibers
Early peak
Dip
Late peak
i
Pre-learning
Post-learning
A/P 0.34
A/P 0.45
Mean ΔF/F
Time (s)
j
ACh-mutant mice light: Post-learning ΔF/F - Pre-learning ΔF/F
Increases (larger peaks or smaller dip post-learning)
Decreases (larger dip or smaller peaks post-learning)
No changes
No component
% of fibers
Number of fibers per mouse
Early peak
Dip
Late peak

Supplementary Fig. 2: Additional quantification of the spatial organization of light and tone cue-evoked ACh release changes during learning. a Top: percent of all fibers with significant increases or decreases, no change, or no significant component from pre to post-learning for the late peak ΔF/F at light cue onset. Bottom: histogram of number of fibers per mouse with significant late peak changes. Each bar is the fiber count for one mouse for each condition indicated by colors at left. **b** Maps (axial, top; sagittal, bottom) showing spatially weighted means across locations of differences with learning (post - pre ΔF/F) for the mean late peak ΔF/F at light cue onset. Lines indicate the axes of maximal variation and arrows indicate the direction of peak increases from smallest to largest changes. White contours indicate regions with changes in the highest 10th percentile. **c** Top: percent of all fibers with significant ΔF/F increases or decreases, no change, or no significant component from pre to post learning for each component at tone cue onset. Bottom: histogram of number of fibers per mouse with significant changes for each component. Each bar is the fiber count for one mouse for each condition indicated by colors at top. **d** Maps (axial, top; sagittal, bottom) showing spatially weighted means across locations of differences with learning (post-pre ΔF/F) for the mean ΔF/F for the three signal components at tone cue onset. Lines indicate the axes of maximal variation and arrows indicate the direction of components increases from smallest to largest changes. White contours indicate regions with changes in the highest 10th percentile. **e** Maps showing the difference in mean light-cue evoked ΔF/F with learning (post – pre) for the three signal components for each fiber (circle). Gray circles, no significant difference; Xs, no significant component. Significance calculated with two-tailed Wilcoxon rank-sum test ($p < 0.01$). **f** Same as (**e**) for tone cue. **g** maps showing each fiber (dot) color coded according to whether significant changes from pre to post learning were present for the light cue onset only (dark blue), tone cue onset only (green) or both (pink). **h** Fraction of all fibers for each component classified according to changes across learning for light cue, tone cue, or both as indicated in (**g**), data are presented as the mean ± SEM, with each dot representing one mouse (n=8), ($p = 0.91$, for early peak vs. dip, Fisher's exact test, two-sided). **i** Mean ΔF/F for 2 representative fibers (same as the fibers shown in Fig. 2f (left) and Fig. 2h) aligned to tone cue onset for trials pre (blue) and post (orange) learning. Shaded regions, S.E.M. Red dots in insets indicate the fiber locations in the coronal plane. **j** Top left: percent of all fibers in mice expressing the non-functional mutant ACh sensor with significant ΔF/F increases or decreases, no change, or no significant component from pre to post learning for each component at light cue onset. Top right: histogram of number of fibers per mouse with significant early peak changes. Each bar is the fiber count for one mouse for each condition indicated by colors at left. Bottom: Maps showing the difference in mean light-cue evoked ΔF/F with learning (post – pre) for the three signal components for each fiber (circle) in mice expressing the mutant ACh sensor. Gray circles, no significant difference; Xs, no significant component. Significance calculated with two-tailed Wilcoxon rank-sum test ($p < 0.01$). Brain schematics in (i) were adapted from the Allen Mouse Brain Common Coordinate Framework (CCFv3) https://atlas.brain-map.org/. Source data are provided as a Source data file.

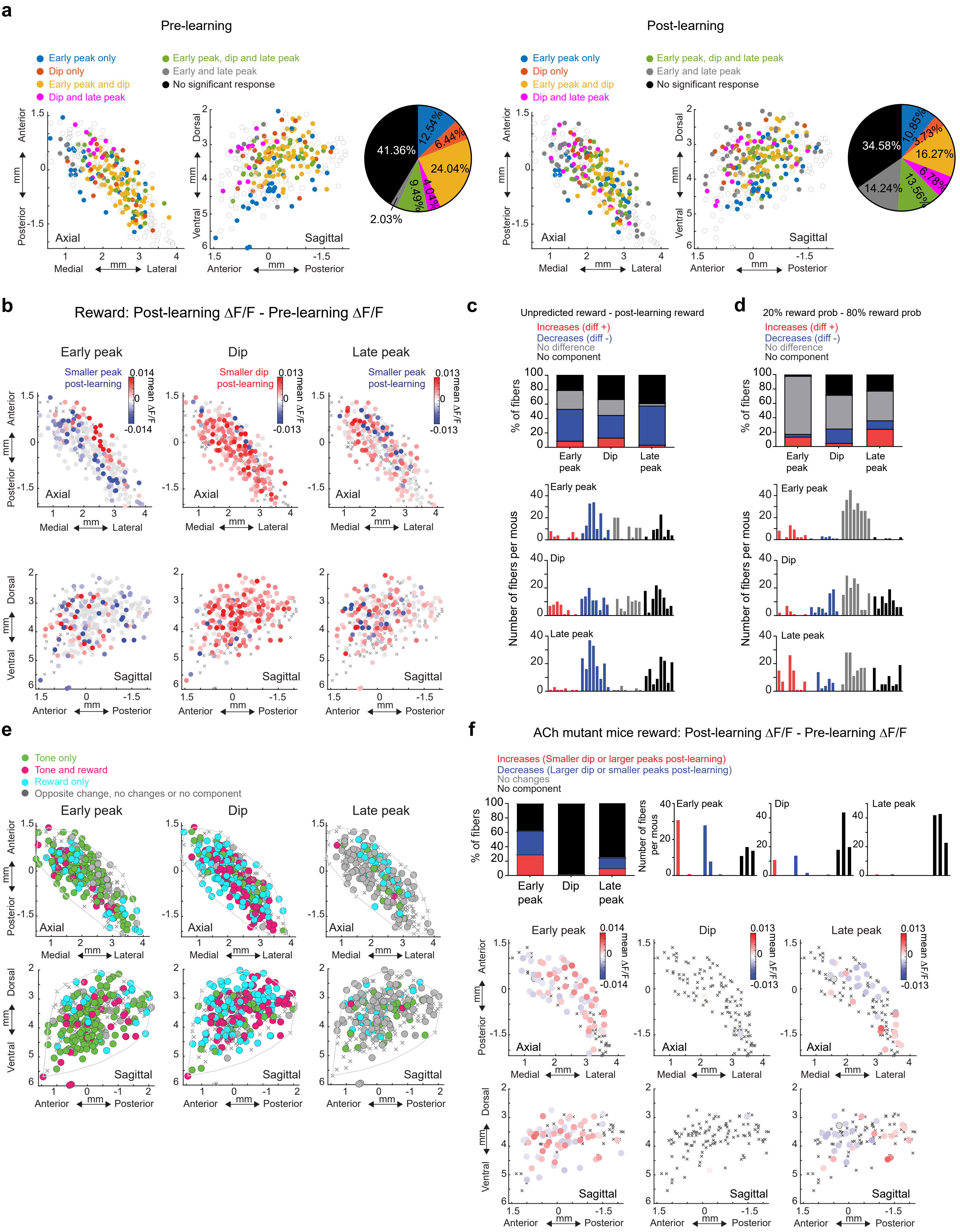

a
Pre-learning
Post-learning
Early peak only
Dip only
Early peak and dip
Dip and late peak
Early peak, dip and late peak
Early and late peak
No significant response
Anterior
Posterior
Medial
Lateral
Dorsal
Ventral
Axial
Sagittal
mm
41.36%
12.54%
6.44%
24.04%
4.04%
9.49%
2.03%
34.58%
10.85%
3.73%
16.27%
6.78%
13.56%
14.24%
b
Reward: Post-learning ΔF/F - Pre-learning ΔF/F
Early peak
Dip
Late peak
Smaller peak post-learning
Smaller dip post-learning
mean ΔF/F
c
Unpredicted reward - post-learning reward
Increases (diff +)
Decreases (diff -)
No difference
No component
% of fibers
Number of fibers per mous
d
20% reward prob - 80% reward prob
e
Tone only
Tone and reward
Reward only
Opposite change, no changes or no component
f
ACh mutant mice reward: Post-learning ΔF/F - Pre-learning ΔF/F
Increases (Smaller dip or larger peaks post-learning)
Decreases (Larger dip or smaller peaks post-learning)
No changes
No component
Number of fibers per mous

Supplementary Fig. 3: Additional quantification of changes in reward-evoked ACh release and mutant ACh-sensor controls during learning. a Maps indicating the presence of each combination of signal components to reward consumption onset for each fiber (circle) pre (left) and post (right) learning. Empty circles are fibers with no significant response. Pie charts indicate percentages of fibers with each combination of significant signal components at reward consumption onset pre (left) and post (right) learning. **b** Maps showing the difference in mean reward consumption evoked ΔF/F with learning (post – pre) for the three signal components for each fiber (circle). Gray circles, no significant difference; Xs, no significant component. Significance calculated with two-tailed Wilcoxon rank-sum test ($p < 0.01$). **c** Top: percent of all fibers with significant ΔF/F differences between unpredicted random rewards and cue predicted rewards post-learning for each component. Bottom: histogram of number of fibers per mouse with significant changes for each component. Each bar is the fiber count for one mouse for each condition indicated by colors at top. **d** Same as (**c**) for ΔF/F differences between rewards following the 20% (less predicted) and 80% (more predicted) cues during the extinction phase. **e** Maps showing each fiber (circle) color coded according to whether significant changes from pre to post learning were present at the tone cue onset only (green), reward consumption only (light blue) or both (pink). Pink dots indicate locations where the component magnitude became larger (dip more negative or peak more positive) for cue and smaller for reward over learning, consistent with reward prediction error encoding. **f** Top left: percent of all fibers in mice expressing the non-functional mutant ACh sensor with significant ΔF/F increases or decreases, no change, or no significant component from pre- to post-learning for each component at reward consumption onset. Top right: histogram of number of fibers per mouse with significant changes. Each bar is the fiber count for one mouse for each condition indicated by colors at left. Note that a larger fraction of artifacts were present for the early peak, but these changes were relatively small, were predominantly present in one mouse, and did not correspond with the primary changes highlighted in the ACh3.0 sensor mice (see Fig. 3). Bottom: Maps as in (**b**) showing the difference in mean reward evoked ΔF/F with learning (post – pre) for the three signal components for each fiber (circle) in mice expressing the mutant ACh sensor. Gray circles, no significant difference; Xs, no significant component. Significance calculated with two-tailed Wilcoxon rank-sum test ($p < 0.01$). Source data are provided as a Source data file.

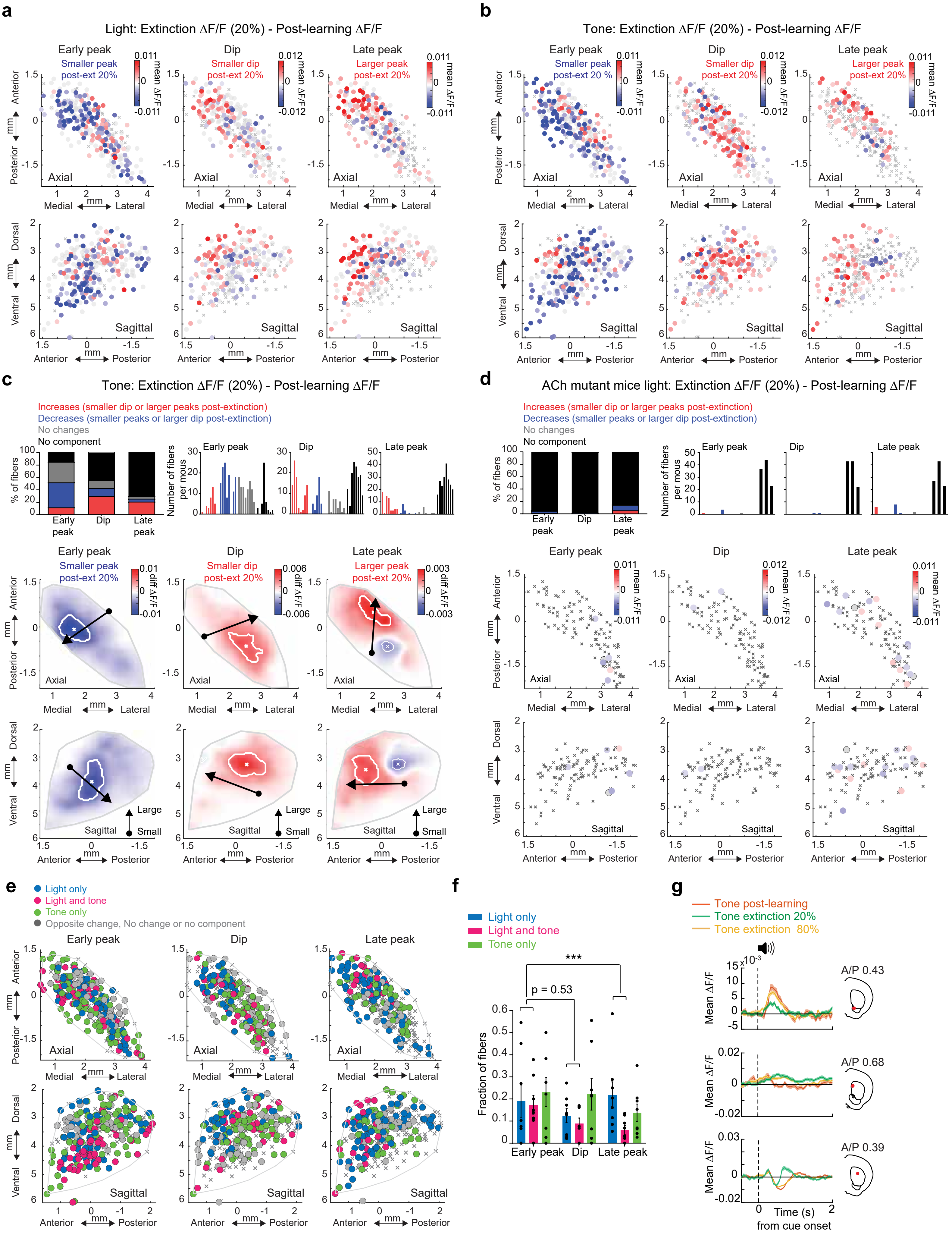
a
Light: Extinction ΔF/F (20%) - Post-learning ΔF/F
Early peak
Smaller peak post-ext 20%
Dip
Smaller dip post-ext 20%
Late peak
Larger peak post-ext 20%
mean ΔF/F
Anterior
Posterior
Axial
Medial
Lateral
Dorsal
Ventral
Sagittal
mm
b
Tone: Extinction ΔF/F (20%) - Post-learning ΔF/F
Early peak
Smaller peak post-ext 20 %
Dip
Smaller dip post-ext 20%
Late peak
Larger peak post-ext 20%
c
Tone: Extinction ΔF/F (20%) - Post-learning ΔF/F
Increases (smaller dip or larger peaks post-extinction)
Decreases (smaller peaks or larger dip post-extinction)
No changes
No component
% of fibers
Number of fibers per mous
Early peak
Dip
Late peak
diff ΔF/F
Large
Small
d
ACh mutant mice light: Extinction ΔF/F (20%) - Post-learning ΔF/F
Increases (smaller dip or larger peaks post-extinction)
Decreases (smaller peaks or larger dip post-extinction)
No changes
No component
e
Light only
Light and tone
Tone only
Opposite change, No change or no component
f
Light only
Light and tone
Tone only
***
p = 0.53
Fraction of fibers
Early peak
Dip
Late peak
g
Tone post-learning
Tone extinction 20%
Tone extinction 80%
Mean ΔF/F
A/P 0.43
A/P 0.68
A/P 0.39
Time (s) from cue onset

Supplementary Fig. 4: Additional quantification of changes in cue-evoked ACh release and mutant ACh-sensor controls during extinction. a Maps showing the difference in mean light cue-evoked ΔF/F between the post-learning (100%) and 20% reward probability phases for the three signal components. Each circle is one fiber. Gray circles, no significant difference; Xs, no significant component. Significance calculated with two-tailed Wilcoxon rank-sum test ($p < 0.01$). **b** Same as (**a**) for tone. **c** Top left: percent of all fibers with significant ΔF/F differences between the 20% extinction and 100% post learning phases (20% - 100% ΔF/F) at tone cue onset for each component. Top right: histogram of number of fibers per mouse with significant changes for each component. Each bar is the fiber count for one mouse for each condition indicated by colors at top. Bottom: Maps (axial, top; sagittal, bottom) showing spatially weighted means across locations of differences between the 20% extinction and 100% post learning phases (20% - 100% ΔF/F) for the mean early peak ΔF/F at tone cue onset. Lines indicate the axes of maximal variation and arrows indicate the direction of peak decreases from smallest to largest changes. White contours indicate regions with changes in the highest 10th percentile. **d** Top: fiber percentages and counts per mouse as in (**c**) for the light cue in mice expressing the non-functional mutant ACh sensor. Bottom: maps as in (**a**) for the mutant ACh sensor mice for the light cue. **e** Maps showing each fiber (circle) color coded according to whether significant differences between the 20% extinction and 100% post learning phases were present for the light cue onset only (dark blue), tone cue onset only (green) or both (pink). **f** Fraction of all fibers for each component classified according to changes across extinction for light cue, tone cue, or both as indicated at top, data are presented as the mean ± SEM, with each dot representing one mouse (n=8), ($p = 0.53$ for early peak vs. dip and $p = 0.0008$ for early peak vs. late peak; Fisher's exact test, two-sided *** $p<0.001$). **g** Mean ΔF/F for 3 representative fibers aligned to tone cue onset for trials where tone was associated with 100% (post-learning), 80%, and 20% (extinction) probabilities. Shaded regions, S.E.M. across trials. Red dots in the insets indicate the fiber locations in the coronal plane. Top and middle examples are the same fibers shown in Fig. 4e and g, respectively). Brain schematics in (g) were adapted from the Allen Mouse Brain Common Coordinate Framework (CCFv3) https://atlas.brain-map.org/. Source data are provided as a Source data file.

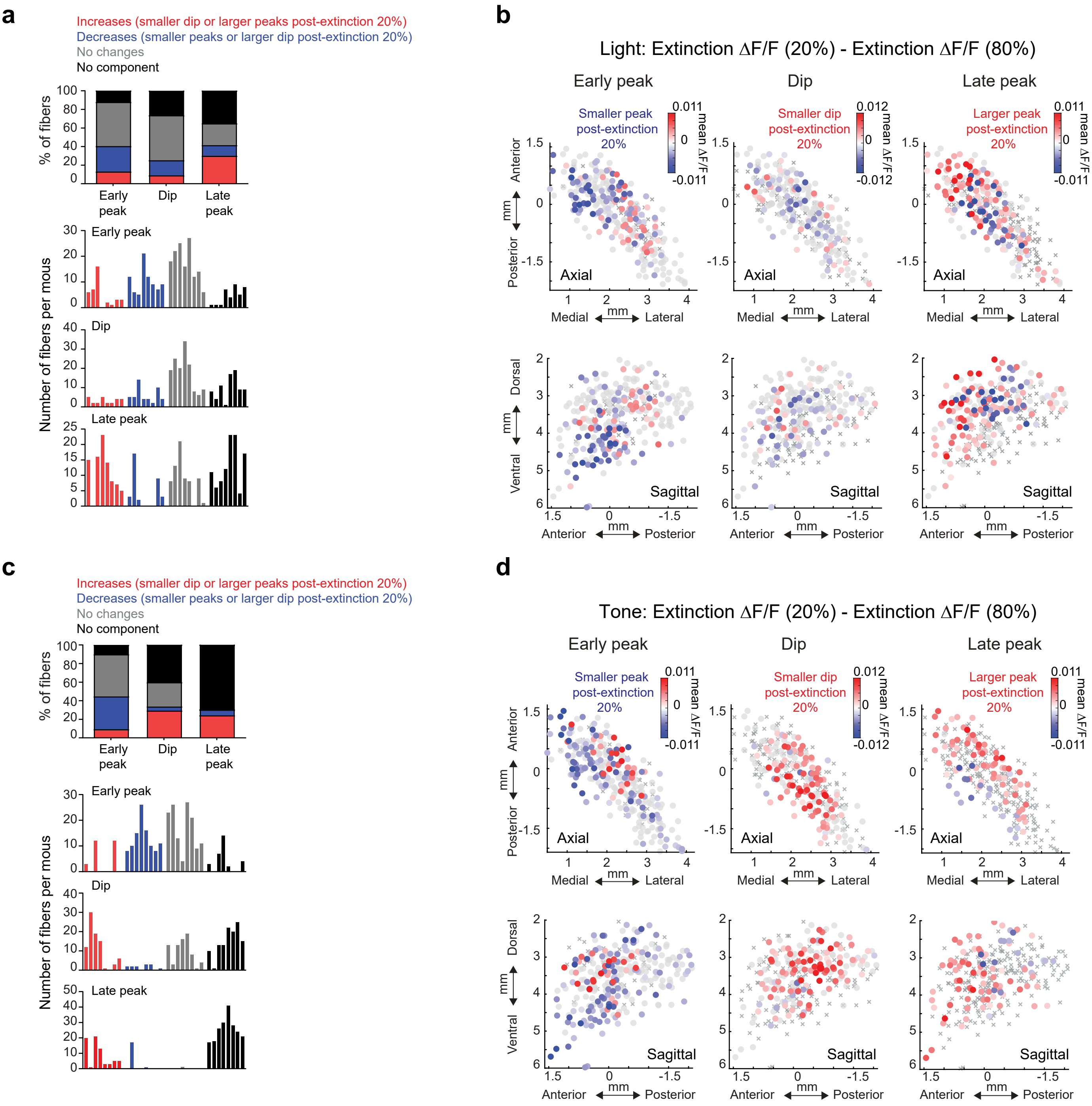
a
Increases (smaller dip or larger peaks post-extinction 20%)
Decreases (smaller peaks or larger dip post-extinction 20%)
No changes
No component
% of fibers
Early peak
Dip
Late peak
Number of fibers per mous
b
Light: Extinction ΔF/F (20%) - Extinction ΔF/F (80%)
Early peak
Dip
Late peak
Smaller peak post-extinction 20%
Smaller dip post-extinction 20%
Larger peak post-extinction 20%
mean ΔF/F
Anterior
Posterior
Medial
Lateral
mm
Axial
Dorsal
Ventral
Sagittal
c
Increases (smaller dip or larger peaks post-extinction 20%)
Decreases (smaller peaks or larger dip post-extinction 20%)
No changes
No component
d
Tone: Extinction ΔF/F (20%) - Extinction ΔF/F (80%)

Supplementary Fig. 5: Comparison of ACh release for high and low probability cues during extinction sessions. a Top: percent of all fibers with significant ΔF/F differences between the 20% extinction and 80% extinction phases (20% - 80% ΔF/F) at light cue onset for each component. Bottom: histogram of number of fibers per mouse with significant changes for each component. Each bar is the fiber count for one mouse for each condition indicated by colors at top. **b** Maps showing the difference in mean light cue-evoked ΔF/F between the 80% and 20% reward probability phases during extinction (20% - 80%) for the three signal components. Each circle is one fiber. Gray circles, no significant difference; Xs, no significant component. Significance calculated with two-tailed Wilcoxon rank-sum test ($p < 0.01$). **c** Percentages and fiber counts per mouse as in (**a**) for ΔF/F differences between the 20% extinction and 80% extinction phases (20% - 80% ΔF/F) at tone cue onset. **d** Maps as in (**b**) for the tone cue. Source data are provided as a Source data file.

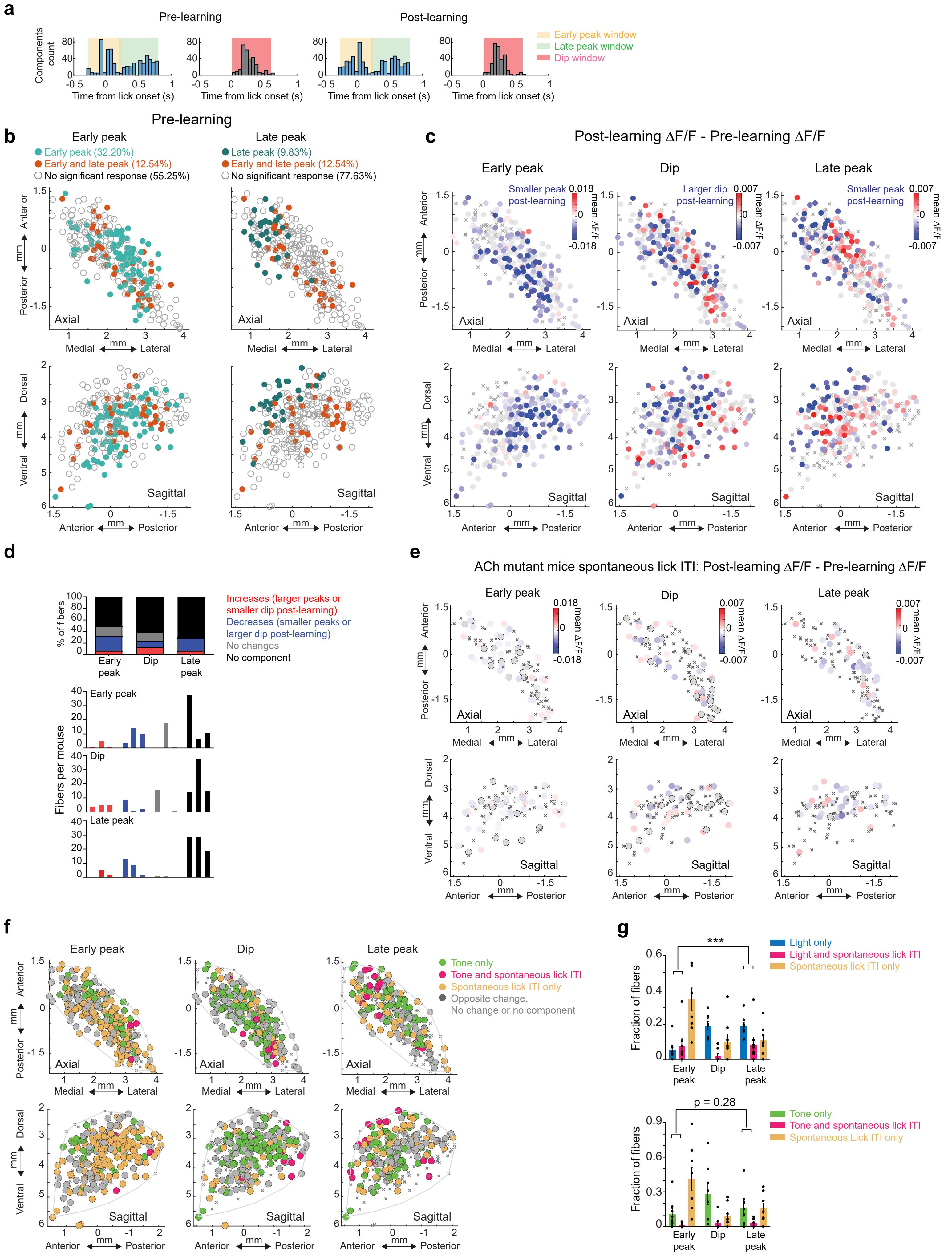

a
Pre-learning
Post-learning
Components count
Time from lick onset (s)
Early peak window
Late peak window
Dip window
b
Pre-learning
Early peak
Late peak
Early peak (32.20%)
Early and late peak (12.54%)
No significant response (55.25%)
Late peak (9.83%)
Early and late peak (12.54%)
No significant response (77.63%)
Anterior
Posterior
Medial
Lateral
Dorsal
Ventral
Axial
Sagittal
mm
c
Post-learning ΔF/F - Pre-learning ΔF/F
Early peak
Dip
Late peak
Smaller peak post-learning
Larger dip post-learning
mean ΔF/F
d
% of fibers
Increases (larger peaks or smaller dip post-learning)
Decreases (smaller peaks or larger dip post-learning)
No changes
No component
Fibers per mouse
e
ACh mutant mice spontaneous lick ITI: Post-learning ΔF/F - Pre-learning ΔF/F
f
Tone only
Tone and spontaneous lick ITI
Spontaneous lick ITI only
Opposite change,
No change or no component
g
Light only
Light and spontaneous lick ITI
Spontaneous lick ITI only
Fraction of fibers
***
p = 0.28
Tone only
Tone and spontaneous lick ITI
Spontaneous Lick ITI only

Supplementary Fig. 6: Additional quantification of changes in unrewarded lick evoked ACh release and mutant ACh-sensor controls during learning. a Histogram of latencies to significant (see Methods) peaks and troughs to unrewarded ITI lick bout onsets pre and post learning. Shaded regions indicate time windows used to identify each component (early peak, dip, and late peak). **b** Maps (axial, left; sagittal, right) indicating the presence of each combination of signal components to unrewarded ITI lick bouts for each fiber (circle) in the pre-learning phase. Empty circles are fibers with no significant response. **c** Maps showing the difference in mean ITI lick-evoked $\Delta F/F$ between the pre and post learning phases (post - pre) for the three signal components. Each circle is one fiber. Gray circles, no significant difference; Xs, no significant component. Significance calculated with two-tailed Wilcoxon rank-sum test ($p < 0.01$). **d** Top: percent of all fibers with significant $\Delta F/F$ differences between the pre and post learning phases at ITI lick onset for each component in mice expressing non-functional mutant ACh sensor. Bottom: histogram of number of fibers per mouse with significant changes for each component. Each bar is the fiber count for one mouse for each condition indicated by colors at top. **e** Same as (**b**) for mice expressing the non-functional mutant ACh sensor. Note that although some sparse artifacts were detected in mutant sensor mice, the spatial organization and magnitude of the artifacts did not match the true signals in the ACh sensor mice (Fig. 5). **f** Maps (axial, top; sagittal, bottom) showing each fiber (dot) color coded according to whether significant changes from pre to post learning (see Methods) were present at the tone cue onset only (green), unrewarded ITI lick only (orange) or both (pink). Pink dots indicate locations where the component magnitude was larger with extinction (dip more negative or peak more positive) for cue and smaller post-learning for the unrewarded ITI lick, consistent with negative reward prediction error encoding. **g** Fraction of all fibers for each component classified according to changes across extinction for light cue (top) or tone cue (bottom) and across learning for unrewarded licks as indicated in (**f**), data are presented as the mean ± SEM, with each dot representing one mouse ($n=8$), $p= 0.0027$ for early peak vs. late peak, $p= 0.28$ for early peak vs. late peak, ***$p <0.001$, Fisher's exact test, two-sided). Source data are provided as a Source data file.

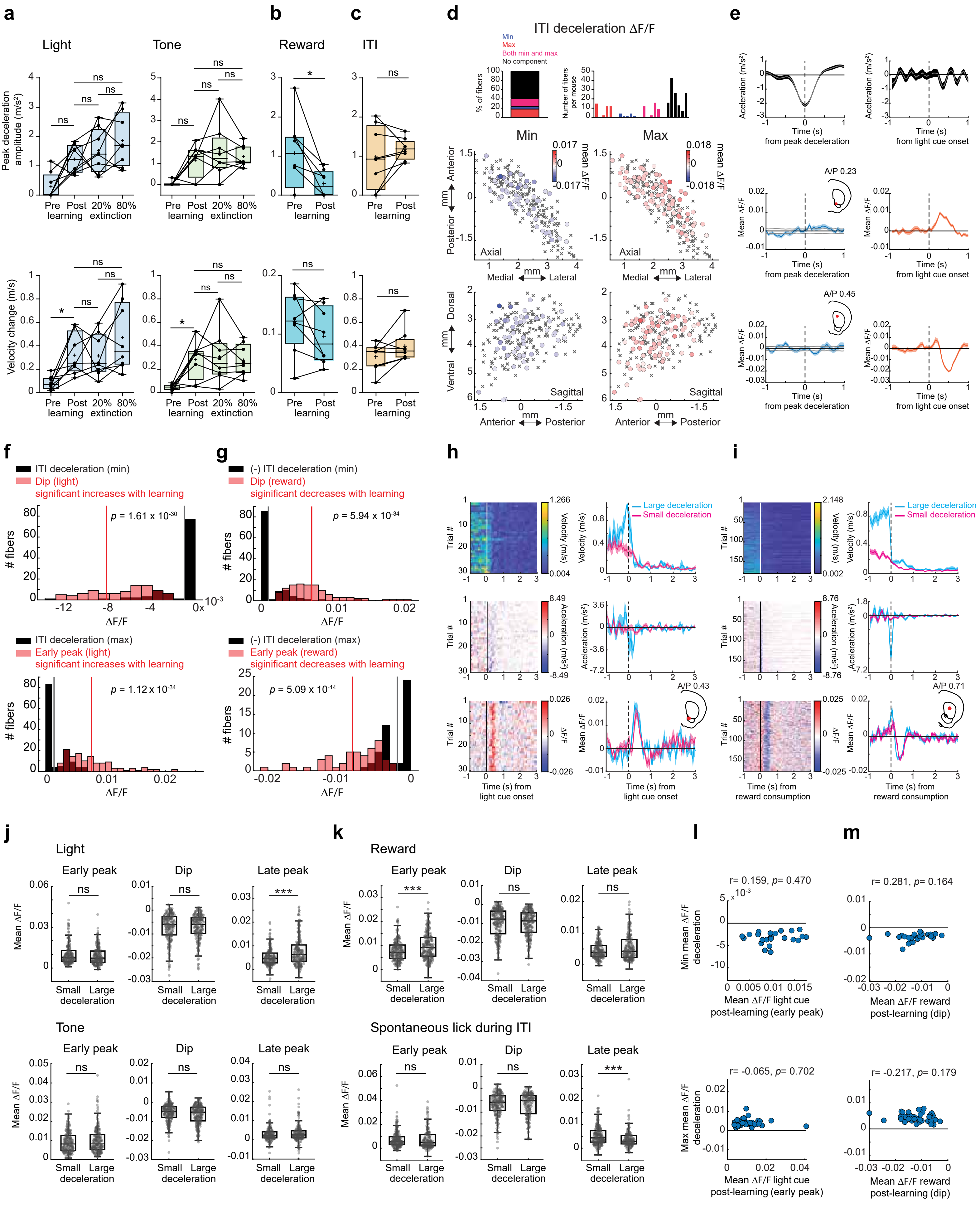

a
Light
Tone
Peak deceleration amplitude (m/s²)
Velocity change (m/s)
Pre learning
Post learning
20% extinction
80% extinction
ns
*
b
Reward
c
ITI
d
ITI deceleration ΔF/F
Min
Max
Both min and max
No component
% of fibers
Number of fibers per mouse
Min
Max
mean ΔF/F
Anterior
Posterior
Axial
Medial
Lateral
Dorsal
Ventral
Sagittal
mm
e
Acceleration (m/s²)
Time (s) from peak deceleration
Time (s) from light cue onset
A/P 0.23
A/P 0.45
Mean ΔF/F
f
ITI deceleration (min)
Dip (light)
significant increases with learning
p = 1.61 x 10^-30
ITI deceleration (max)
Early peak (light)
significant increases with learning
p = 1.12 x 10^-34
# fibers
ΔF/F
g
(-) ITI deceleration (min)
Dip (reward)
significant decreases with learning
p = 5.94 x 10^-34
(-) ITI deceleration (max)
Early peak (reward)
significant decreases with learning
p = 5.09 x 10^-14
h
Trial #
Velocity (m/s)
Acceleration (m/s²)
Large deceleration
Small deceleration
A/P 0.43
Time (s) from light cue onset
i
A/P 0.71
Time (s) from reward consumption
j
Light
Early peak
Dip
Late peak
Tone
Mean ΔF/F
Small Large deceleration
ns
***
k
Reward
Spontaneous lick during ITI
l
r= 0.159, p= 0.470
r= -0.065, p= 0.702
Min mean ΔF/F deceleration
Max mean ΔF/F deceleration
Mean ΔF/F light cue post-learning (early peak)
m
r= 0.281, p= 0.164
r= -0.217, p= 0.179
Mean ΔF/F reward post-learning (dip)

Supplementary Fig. 7: Learning and extinction related changes in striatum-wide ACh release cannot be explained by locomotion changes. **a** Box and whisker plots showing average peak decelerations (top) and velocity changes (bottom) across all mice (n=8 mice) following light (left) tone (right) cue onsets for each learning phase. (n.s., not significant Friedman test, p >0.05, p = 0.04 for light and tone velocity, *p<0.05). Each datapoint is the mean for one mouse. **b** Same as (**a**) for reward delivery pre and post learning (Two-tailed Wilcoxon rank-sum test, **p* = 0.03 for peak deceleration and p = 0.14 for velocity; p<0.05). **c** Same as (**b**) for unrewarded lick bout onsets in the ITI (Two-tailed Wilcoxon test, p = 0.38 for peak deceleration and p = 0.19 for velocity). Box bounds indicate the 25th and 75th percentiles and the whiskers represent the minima and maxima. The center line represents the median. **d** Top left: Percent of all fibers with significant minima, maxima, or both (min and max) of the mean ACh ΔF/F within a ±1-s window triggered on troughs of large spontaneous decelerations in the intertrial interval (ITI). Top right: Histogram of the number of fibers per mouse with significant changes for minima, maxima, or both. Bottom: Maps (axial, top; sagittal, bottom) showing the minima (left) and maxima (right) of the mean ACh ΔF/F triggered on troughs of large ITI decelerations. Significance for each fiber (circle) was determined by comparison to the 95% confidence interval of a bootstrapped ΔF/F distribution (Methods). Xs, no significant response. **e** Left column: Mean acceleration (top) and mean ΔF/F (middle and bottom) aligned to ITI deceleration troughs for 2 representative fibers with small but significant minima and maxima. Shaded regions, S.E.M. Gray shading indicates the 95 % confidence interval. Insets indicate fiber location in the coronal plane. Right column: Mean acceleration (top) and mean ΔF/F (middle and bottom) aligned to light cue onset for trials post-learning for the same representative fibers shown in the left column. Shaded region, S.E.M. **f** Red: Histogram showing the magnitudes of ΔF/F changes from pre to post learning for the dip (top) and early peak (bottom) components for the light cue for fibers with significant changes. Black: Histogram of the magnitudes of the ΔF/F minima (top) and maxima (bottom) of the spontaneous ITI deceleration triggered ΔF/F for the same fibers with changes over learning plotted in red. Fibers with no significant deceleration ITI response counted in the 0 bin. Lines indicate means (Two-tailed Wilcoxon rank-sum test). **g** Same as (**f**) for reward delivery periods. The sign of the ITI deceleration response magnitudes is flipped for visualization because the deceleration magnitudes to reward delivery decrease with learning, opposite to cues. **h** Left column: Total treadmill velocity (top), acceleration (middle), and ΔF/F (bottom) aligned to light cue onsets for all trials post-learning for a single example fiber with a significant early peak increase with learning sorted by the deceleration magnitude following cue onset. Right column: Mean cue-triggered averages for measures in left column for trials with large decelerations (>80th percentile of decelerations from the distribution of decelerations across all mice, n = 4 trials, blue) and small decelerations (<20th percentile, n = 8 trials, magenta). Shaded region, S.E.M**.** **i** Same as (**h**) but aligned on reward consumption for another example fiber with a significant change in the reward dip magnitude with learning. (n = 24 and 31 trials for large and small deceleration trials respectively). **j** Box and whisker plots showing the mean light (top) and tone (bottom) cue-evoked ΔF/F for each component (early peak, dip and late peak) for trials with small and large decelerations across all fibers (dots). Box bounds indicate the 25th and 75th percentiles and the whiskers represent the minima and maxima, the center line represents the median. Significance calculated between large and small decelerations for each component across fibers using a two tailed Wilcoxon rank-sum test. ***: p< 0.001. n.s.: not significant. **k** same as (**j**) for reward (top) and spontaneous ITI licks (bottom). **l** Scatterplots of the minimum (top) and maximum (bottom) of the mean ΔF/F triggered on ITI deceleration troughs and the mean post-learning light cue-evoked early peak ΔF/F for all fibers (dots) with significant components for both cues and ITI decelerations. R and p values are from Pearson's correlations. **m** same as (**l**) but for the dip component during reward post-learning. The exact p-values and additional statistical details can be found in Supplementary Table 1. Brain schematics in (e), (h) and (i) were adapted from the Allen Mouse Brain Common Coordinate Framework (CCFv3) https://atlas.brain-map.org/. Source data are provided as a Source data file.

<u>Supplementary table 1:</u> Summary of statistical analysis

**Figure**	**Test**	**Test Value**	**Multiple comparison and p values**
**Figure 2**			
2l left	Two-tailed Wald t-test followed by Bonferroni post hoc analysis (Early Peak: 226 fibers, Dip: 196 fibers across 8 mice). Spatial coordinates (AP, ML, DV) were included as fixed effects, while mouse identity was treated as a random effect to account for variability between subjects.	<u>Early peak:</u><br>DF = 222, tStat (AP= 3.5969, ML = 0.9797, DV = 2.2618<br><u>Dip:</u><br>DF = 192, tStat (AP=5.5296, ML = 1.7133, DV = -1.6623)<br><u>Early peak vs dip:</u><br>DF = 414, tStat (AP = 1.948, ML = 1.1417, DV = -3.337).	Bonferroni post hoc <u>Light</u><br><u>Early peak</u> (**AP:** P =0.0011, **ML:** P =0.9848, **DV:** P =0.0740)<br><u>Dip</u> (**AP:** P =3.1161 x 10^{-7}, **ML:** P = 0.2648, **DV:** P =0.2942)<br><u>Early peak vs dip</u> (**AP:** P =0.1562, **ML:** P =0.7626, **DV:** P =0.0027)
2l right	Two-tailed Wald t-test followed by Bonferroni post hoc analysis (Early Peak: 218 fibers, Dip: 130 fibers across 8 mice). Spatial coordinates (AP, ML, DV) were included as fixed effects, while mouse identity was treated as a random effect to account for variability between subjects.	<u>Early peak:</u><br>DF = 214, tStat (AP= 1.3943, ML = -0.36018, DV = 1.9176<br><u>Dip:</u><br>DF = 126, tStat (AP=5.1805, ML = 4.9999, DV = -0.40445)<br><u>Early peak vs dip:</u><br>DF = 340, tStat (AP = 5.237, ML = 6.6595, DV = -1.049).	Bonferroni post hoc <u>Tone</u><br><u>Early peak</u> (**AP:** P = 0.4940, **ML:** P =2.1572, **DV:** P =0.1694)<br><u>Dip</u> (**AP:** P =2.559 x 10^{-6}, **ML:** P =5.635x 10^{-6}, **DV:** P =2.0596)<br><u>Early peak vs dip</u> (**AP:** P =8.559 x 10^{-7}, **ML:** P =3.318 x 10^{-10}, **DV:** P =0.8847)
2m left	Two-tailed Wald t-test followed by Bonferroni post hoc analysis (Early peak light: 226 fibers, early peak tone: 218 fibers across 8 mice). Spatial coordinates (AP, ML, DV) were included as fixed effects, while mouse identity was treated as a random effect to account for variability between subjects.	<u>Light:</u><br>DF = 222, tStat (AP = 3.5969, ML=0.9797, DV= 2.2618)<br><br><u>Tone:</u><br>DF = 126, tStat (AP = 1.3943, ML = -0.36018, DV = 1.9176)<br><br><u>Light vs Tone:</u><br>DF = 436, tStat (AP = -2.8373, ML = -2.4367, DV = -0.24076)	Bonferroni post hoc <u>Early peak</u><br><u>Light</u> (**AP:** P=0.0011, **ML:** P=0.9848, **DV:** P=0.0740)<br><u>Tone</u> (**AP:** P=0.4940, **ML:** P=2.1572, **DV:** P=0.1694)<br><u>Light vs Tone</u> (**AP:** P=0.014, **ML:** P=0.045, **DV:** P=2.42955)

2m middle	Two-tailed Wald t-test followed by Bonferroni post hoc analysis (Dip light: 196 fibers, dip tone: 130 fibers across 8 mice). Spatial coordinates (AP, ML, DV) were included as fixed effects, while mouse identity was treated as a random effect to account for variability between subjects.	Light:<br>DF = 192, tStat (AP = 5.5296, ML = 1.7133, DV = -1.6623)<br>Tone:<br>DF = 126, tStat (AP = 5.1805, ML = 4.9999, DV = -0.40445)<br>Light vs Tone:<br>DF = 318, tStat (AP = 1.8899, ML = 3.9443, DV = 1.3186)	Bonferroni post hoc Dip<br>Light (**AP:** P=3.1161 x 10^{-7}, **ML:** P=0.2648, **DV:** P=0.2942)<br>Tone (**AP:** P=2.559 x 10^{-6}, **ML:** P=5.635 x 10^{-6}, **DV:** P=2.0596)<br>Light vs Tone (**AP:** P=0.1790, **ML:** P = 0.00029, **DV:** P=0.5647)
2m right	Two-tailed Wald t-test followed by Bonferroni post hoc analysis (Late peak light: 126 fibers, late peak tone: 35 fibers across 8 mice). Spatial coordinates (AP, ML, DV) were included as fixed effects, while mouse identity was treated as a random effect to account for variability between subjects.	Light:<br>DF = 122, tStat (AP = 4.353, ML = 3.7616, DV = -2.1708)<br>Tone:<br>DF = 31, tStat (AP = -0.51275, ML = -0.79185, DV = 1.9751)<br>Light vs Tone:<br>DF = 153, tStat (AP = -2.5898, ML = -2.8501, DV = 0.29465)	Bonferroni post hoc Late Peak<br>Light (**AP:** P=8.4281 x 10^{-5}, **ML:** P=0.00078, **DV:** P=0.095)<br>Tone (**AP:** P=1.8352, **ML:** P=1.3033, **DV:** P=0.1716)<br>Light vs Tone (**AP:** P=0.0315, **ML:** P=0.0149, **DV:** P=2.3059)
**Figure 4**			
4d	RM one-way ANOVA (n=8 mice)	F= 43.61, P<0.0001<br>DF= 5, $F_{(1.693, 11.85)}$ = 43.61, P<0.0001 (between learning phases)<br>DF = 7, $F_{(7, 35)}$ = 4.038, P = 0.0024 (between subjects)	Turkey post hoc<br>L 100% vs L 20%, P = 0.0002<br>T 100% vs L 20%, P = 0.0003<br>L20% vs T80%, P= 0.0407<br>T80% vs T 20%, P= 0.0250<br>T 20% vs L 80%, P <0.0001<br>L20% vs L 80%, P <0.0001<br>T100% vs T 20%, P= 0.0004<br>L100% vs T20%, P= 0.0002
4k left	Two-tailed Wald t-test followed by Bonferroni post hoc analysis (Early peak: 259 fibers, late peak: 190 fibers across 8	Early peak:<br>DF = 255, tStat (AP = -1.9612, ML = -3.5294, DV = 2.6871)<br>Late peak:	Bonferroni post hoc Light<br>Early peak (**AP:** P=0.1528, **ML:** P=0.00148, **DV:** P = 0.02304)<br>Late peak (**AP:** P=2.1851, **ML:** P=0.274, **DV:** P=1.79888)

	mice). Spatial coordinates (AP, ML, DV) were included as fixed effects, while mouse identity was treated as a random effect to account for variability between subjects.	DF = 186, tStat (AP = 0.3478, ML = -1.6972, DV = -0.52584<br>Early peak vs Late peak:<br>DF = 441, tStat (AP = 1.9904, ML = 0.80701, DV = -3.4528)	Early peak vs late peak (**AP:** P=0.0711, **ML:** P=5x10^{-7}, **DV:** P=0.00539)
4k right	Two-tailed Wald t-test followed by Bonferroni post hoc analysis (Early peak: 251 fibers, late peak: 86 fibers across 8 mice). Spatial coordinates (AP, ML, DV) were included as fixed effects, while mouse identity was treated as a random effect to account for variability between subjects.	Early peak:<br>DF = 247, tStat (AP = -2.6737, ML = -3.1826, DV = 4.5184)<br>Late peak:<br>DF = 82, tStat (AP = 1.8878, ML = 0.11924, DV = 0.031266)<br>Early peak vs Late peak:<br>DF = 329, tStat (AP = 4.2149, ML = 3.3039, DV = -3.5093)	Bonferroni post hoc Tone<br>Early peak (**AP:** P=0.024, **ML:** P=0.0494, **DV:** P =2.9x10^{-5})<br>Late peak peak (**AP:** P=0.1877, **ML:** P=2.71614, **DV:** P =2.9254)<br>Early peak vs late peak (**AP:** P=9.69 x 10^{-5}, **ML:** P=0.00318, **DV:** P =0.00154)
4l left	Two-tailed Wald t-test followed by Bonferroni post hoc analysis (Early peak light: 259 fibers, early peak tone: 251 fibers across 8 mice). Spatial coordinates (AP, ML, DV) were included as fixed effects, while mouse identity was treated as a random effect to account for variability between subjects.	Light:<br>DF = 255, tStat (AP = -1.9612, ML = -3.5294, DV = 2.6871)<br>Tone:<br>DF = 247, tStat (AP = -2.6737, ML = -3.1826, DV = 4.5184)<br>Light vs Tone:<br>DF = 502, tStat (AP = -0.76918, ML = -0.48461, DV = 0.34293)	Bonferroni post hoc Early peak<br>Light (**AP:** P=0.1528, **ML:** P = 0.00148, **DV:** P=0.0230)<br>Tone (**AP:** P=0.0240, **ML:** P=0.0049, **DV:** P=2.898x10^{-5})<br>Light vs Tone (**AP:** P=1.3264, **ML:** P=1.8844, **DV:** P=2.1953)
4l middle	Two-tailed Wald t-test followed by Bonferroni post hoc analysis (Dip light: 218 fibers, dip tone: 165 fibers across 8 mice). Spatial	Light:<br>DF = 214, tStat (AP = 0.06061, ML = -2.3731, DV = -4.1343)<br>Tone:	Bonferroni post hoc Dip<br>Light (**AP:** P=2.8551, **ML:** P=0.0555, **DV:** P=0.000153)<br>Tone (**AP:** P=1.12505, **ML:** P=0.1577, **DV:** P=1.4071)

	coordinates (AP, ML, DV) were included as fixed effects, while mouse identity was treated as a random effect to account for variability between subjects.	DF = 161, tStat (AP = 0.88958, ML = 1.9529, DV = -0.72574)<br>Light vs Tone:<br>DF = 375, tStat (AP = 1.0815, ML = 4.4072, DV = 3.1138)	Light vs Tone (**AP:** P=0.84055, **ML:** P=4.1072 x 10^{-5}, **DV:** P=0.00596)
4I right	Two-tailed Wald t-test followed by Bonferroni post hoc analysis (Late peak light: 190 fibers, late peak tone: 86 fibers across 8 mice). Spatial coordinates (AP, ML, DV) were included as fixed effects, while mouse identity was treated as a random effect to account for variability between subjects**.**	Light:<br>DF = 186, tStat (AP = 0.3478, ML = -1.6972, DV = -0.52584)<br>Tone:<br>DF = 82, tStat (AP = 1.8878, ML = 0.11924, DV = 0.031266)<br>Light vs Tone:<br>DF = 268, tStat (AP = 1.5874, ML = 1.9787, DV = 0.76078)	Bonferroni post hoc Late peak Light (**AP:** P=2.1851, **ML:** P=0.2740, **DV:** P=1.7988)<br>Tone (**AP:** P=0.1877, **ML:** P=2.7161, **DV:** P=2.9253)<br>Light vs Tone (**AP:** P=0.3408, **ML:** P=0.1466, **DV:** P=1.3423)
**Figure 6**			
6d	Two-way RM ANOVA<br>Control mice (n=8)<br>TelC mice (n=6)	Learning phases x groups ($F_{(2, 24)}$ = 2.175, P = 0.1355)<br>Learning phases ($F_{(1.816, 21.79)}$ = 25.25, P<0.0001)<br>Groups ($F_{(1, 12)}$ = 4.553, P = 0.0542)<br>Subjects ($F_{(12, 24)}$ = 5.116, P = 0.0003)	Turkey post hoc<br>Between control:<br>Pre- vs post-learning, P = 0.0001<br>Pre- vs Extinction 20%, P = 0.0949<br>Post learning vs extinction 20%, P = 0.0392<br>Between TelC:<br>Pre- vs post-learning, P = 0.0189<br>Pre- vs Extinction 20%, P = 0.0592<br>Post learning vs extinction 20%, P = 0.9290<br>Within:<br>Pre-learning<br>Control vs TelC, P = 0.0426<br>Post-learning<br>Control vs TelC, P = 0.4181<br>Extinction 20%<br>Control vs TelC, P = 0.0627

6e	Two-way RM ANOVA<br>Control mice (n=8)<br>TelC mice (n=6)	Learning phases x groups ($F_{(2, 24)}$ = 0.7993, P = 0.4613)<br>Learning phases ($F_{(1.656, 19.87)}$ = 0.1484, P=0.8244)<br>Groups ($F_{(1, 12)}$ = 3.678, P = 0.0793)<br>Subjects ($F_{(12, 24)}$ = 3.350, P = 0.0057)	Turkey post hoc, No significant differences found<br>Between control:<br>Pre- vs post-learning, P = 0.8072<br>Pre-learning vs Extinction 20%, P = 0.8822<br>Post learning vs extinction 20%, P = 0.6416<br>Between TelC:<br>Pre- vs post-learning, P = 0.9367<br>Pre-learning vs Extinction 20%, P = 0.6454<br>Post learning vs extinction 20%, P = 0.2587<br>Within:<br>Pre-learning<br>Control vs TelC, P = 0.1179<br>Post-learning<br>Control vs TelC, P = 0.3163<br>Extinction 20%<br>Control vs TelC, P = 0.1033
**Figure 7**			
7g left top	Friedman test, two-tailed<br>(N=4 mice)	χ^2 (2) = 0.50, p = 0.93	Dunn's post hoc, No significant differences found<br>Post learning vs. extinction 10%, P > 0.9999<br>Post learning vs. extinction 90%, P > 0.9999<br>Extinction 10% vs 90%, P > 0.9999
7g left bottom	Friedman test, two-tailed<br>(N=4 mice)	χ^2 (2) = 1.5, p = 0.6528	Dunn's post hoc, No significant differences found<br>Post learning vs. extinction 10%, P > 0.8665<br>Post learning vs. extinction 90%, P > 0.8665<br>Extinction 10% vs 90%, P > 0.9999
7g right top	Friedman test, two-tailed<br>(N=4 mice)	χ^2 (2) = 0, p >0.9999	Dunn's post hoc, No significant differences found<br>Post learning vs. extinction 10%, P > 0.9999<br>Post learning vs. extinction 90%, P > 0.9999<br>Extinction 10% vs 90%, P > 0.9999

7g right bottom	Friedman test, two-tailed (N=4 mice)	χ² (2) = 1.5, p = 0.6528	Dunn's post hoc, No significant differences found Post learning vs. extinction 10%, P > 0.8665 Post learning vs. extinction 90%, P > 0.9999 Extinction 10% vs 90%, P > 0.8665
**Supplementary Figure 7**			
7a top left	Friedman test, two-tailed (N=8 mice)	χ² (3) = 14.55, p = 0.0022	Dunn's post hoc, No significant differences found between Pre vs post learning, P = 0.3168 Post learning vs extinction 20%, P>0.9999 Post learning vs extinction 80%, P>0.9999 Extinction 20% vs 80%, P>0.9999 Significant differences were found between Pre learning vs extinction 20%, P = 0.006 Pre learning vs extinction 80%, P = 0.006
7a bottom left	Friedman test, two-tailed (N=8 mice)	χ² (3) = 15.45, p = 0.0015	Dunn's post hoc, significant differences were found between Pre learning vs post, P = 0.0402 Pre learning vs extinction 20%, P = 0.0221 Pre learning vs extinction 80%, P = 0.0014 Dunn's post hoc, no significant differences found between Post learning vs extinction 20%, P>0.9999 Post learning vs extinction 80%, P>0.9999 Extinction 20% vs 80%, P>0.9999
7a top right	Friedman test, two-tailed (N=8 mice)	χ² (3) = 9.423, p = 0.0242	Dunn's post hoc, no significant differences found between

			Pre learning vs post, P = 0.1557<br>Pre learning vs extinction 20%, P = 0.0709<br>Pre learning vs extinction 80%, P = 0.0537<br>Post learning vs extinction 20%, P>0.9999<br>Post learning vs extinction 80%, P>0.9999<br>Extinction 20% vs 80%, P>0.9999
7a bottom right	Friedman test, two-tailed<br>(N=8 mice)	χ^2 (3) = 10.65, p = 0.0138	Dunn's post hoc, significant differences found between<br>Pre learning vs post, P = 0.0402<br>No significant differences found between<br>Pre learning vs extinction 20%, P = 0.1990<br>Significant differences found between<br>Pre learning vs extinction 80%, P = 0.0221<br>No significant differences found between<br>Post learning vs extinction 20%, P>0.9999<br>Post learning vs extinction 80%, P>0.9999<br>Extinction 20% vs 80%, P>0.9999
7b top	Wilcoxon Matched-Pairs Signed-Rank Test, Two-tailed<br>(N=8 mice)	W = -21, P = 0.0312	
7b bottom	Wilcoxon Matched-Pairs Signed-Rank Test, Two-tailed<br>(N=8 mice)	W = -22, P = 0.1484	
7c top	Wilcoxon Matched-Pairs Signed-Rank Test, Two-tailed<br>(N=8 mice)	W = 14, P = 0.3828	
7c bottom	Wilcoxon Matched-Pairs Signed-Rank Test, Two-tailed<br>(N=8 mice)	W = 20, P = 0.1953	

7j top	Two-tailed Wilcoxon rank sum test	Early peak: (n=222 fibers for small and large decelerations)<br>W = 51892, zStat = 1.84666, P = 0.0647<br>Dip: (n=222 fibers for small and large decelerations)<br>W = 50103, zStat = 0.5233, P = 0.60073<br>Late peak: (n=191 fibers for small decelerations and n = 201 fibers for large decelerations)<br>W = 33197, zStat = -3.86516, P = 0.000111	
7j bottom	Two-tailed Wilcoxon rank sum test	Early peak: (n=222 fibers for small and large decelerations)<br>W = 48206, zStat = -0.87913, P = 0.37932<br>Dip: (n=222 fibers for small and large decelerations)<br>W = 51752, zStat = 1.74311, P = 0.081314<br>Late peak: (n=192 fibers for small decelerations and n = 188 fibers for large decelerations)<br>W = 35834, zStat = -0.69264, P = 0.48853	
7k top	Two-tailed Wilcoxon rank sum test	Early peak: (n=222 fibers for small and large decelerations)<br>W =44307, zStat =-3.76324, P = 0.00016<br>Dip: (n=222 fibers for small and large decelerations)<br>W =50446, zStat =0.77705, P = 0.4371<br>Late peak: (n=201 fibers for small decelerations and n = 212 fibers for large decelerations)<br>W =39939, zStat =-1.37527, P = 0.16904	
7k bottom	Two-tailed Wilcoxon rank sum test	Early peak: (n = 219 fibers for small decelerations, and	

		n=176 fibers for large decelerations)<br>W =44923, zStat =-1.38365, P = 0.16643<br>Dip: (n = 222 fibers for small decelerations, and n=176 fibers for large decelerations)<br>W =44563, zStat =0.23995, P = 0.81036<br>Late peak: (n = 219 fibers for small decelerations, and n=177 fibers for large decelerations)<br>W =48473, zStat =-4.41614, P = 1.004×10^{-5}	

Supplementary table 2: Pearson's correlation coefficients and two-tailed p-values between the minimum and maximum of the mean ΔF/F triggered on ITI deceleration troughs and the mean cue or reward evoked ΔF/F for the three components for pre and post learning phases. Correlations are limited to fibers with both significant deceleration and task-related changes for each component. Empty cells are conditions with fewer than 10 fibers.

Supplementary table 2: Pearson's correlation of minimum and maximum mean ΔF/F of ITI deceleration with cue and reward component signals							
		Early Peak		Dip		Late peak	
		r	*p*-value	r	*p*-value	r	*p*-value
Light pre-learning	Min						
	Max						
Light post-learning	Min	0.159	0.47	0.497	0.042	0.315	0.346
	Max	-0.065	0.702	-0.159	0.429	0.307	0.201
Tone pre-learning	Min						
	Max	0.398	0.178	0.868	0.056		
Tone post-learning	Min	-0.324	0.093	0.074	0.802		
	Max	0.245	0.097	-0.565	0.006		
Reward pre-learning	Min	0.022	0.923	-0.342	0.094	-0.274	0.242
	Max	-0.022	0.902	-0.325	0.038	0.088	0.668
Reward post-learning	Min	0.292	0.24	0.281	0.164	0.332	0.113
	Max	0.092	0.61	-0.217	0.179	-0.058	0.737
