# Supplementary material for: Distinct spatially organized striatum-wide acetylcholine dynamics for the learning and extinction of Pavlovian associations
Source: Nat Commun. 2025 Jun 4;16:5169. doi: 10.1038/s41467-025-60462-5 (PMC12137636; doi:10.1038/s41467-025-60462-5)
Supplement: Supplementary file 2 — Reporting Summary [file 41467_2025_60462_MOESM2_ESM.pdf]

nature portfolio

Corresponding author(s): Mark Howe

Last updated by author(s): Apr 29, 2025

Reporting Summary

Nature Portfolio wishes to improve the reproducibility of the work that we publish. This form provides structure for consistency and transparency in reporting. For further information on Nature Portfolio policies, see our Editorial Policies and the Editorial Policy Checklist.

Statistics

For all statistical analyses, confirm that the following items are present in the figure legend, table legend, main text, or Methods section.

n/a	Confirmed	
☐	☒	The exact sample size (*n*) for each experimental group/condition, given as a discrete number and unit of measurement
☐	☒	A statement on whether measurements were taken from distinct samples or whether the same sample was measured repeatedly
☐	☒	The statistical test(s) used AND whether they are one- or two-sided<br>*Only common tests should be described solely by name; describe more complex techniques in the Methods section.*
☐	☒	A description of all covariates tested
☐	☒	A description of any assumptions or corrections, such as tests of normality and adjustment for multiple comparisons
☐	☒	A full description of the statistical parameters including central tendency (e.g. means) or other basic estimates (e.g. regression coefficient) AND variation (e.g. standard deviation) or associated estimates of uncertainty (e.g. confidence intervals)
☐	☒	For null hypothesis testing, the test statistic (e.g. *F*, *t*, *r*) with confidence intervals, effect sizes, degrees of freedom and *P* value noted<br>*Give P values as exact values whenever suitable.*
☒	☐	For Bayesian analysis, information on the choice of priors and Markov chain Monte Carlo settings
☒	☐	For hierarchical and complex designs, identification of the appropriate level for tests and full reporting of outcomes
☐	☒	Estimates of effect sizes (e.g. Cohen's *d*, Pearson's *r*), indicating how they were calculated

Our web collection on statistics for biologists contains articles on many of the points above.

Software and code

Policy information about availability of computer code

Data collection	Photometry data were acquired using HCImage Live software (RRID:SCR_015041), behavior data were collected using custom code implemented in Matlab 2017b (RRID:SCR_001622)
Data analysis	Photometry data were analyzed using a combination of custom and publicly available code (Matlab2020b, 2022b and 2023; RRID:SCR_001622). A subset of behavioral data was analyzed using Graphpad Prism 10 (RRID:SCR_002798)<br><br>Public use versions of our pipeline and code for photometry imaging data preprocessing and CT-based fiber localization are available on GitHub at: https://github.com/HoweLab/MultifiberProcessing and https://github.com/HoweLab/MultifiberLocalization. The custom code for data analysis is available on https://github.com/craggASAP/Pavlovian_Associations.git.

For manuscripts utilizing custom algorithms or software that are central to the research but not yet described in published literature, software must be made available to editors and reviewers. We strongly encourage code deposition in a community repository (e.g. GitHub). See the Nature Portfolio guidelines for submitting code & software for further information.

Data

Policy information about availability of data

All manuscripts must include a data availability statement. This statement should provide the following information, where applicable:

- Accession codes, unique identifiers, or web links for publicly available datasets
- A description of any restrictions on data availability
- For clinical datasets or third party data, please ensure that the statement adheres to our policy

The datasets generated in this study have been deposited in the Zenodo database under accession code 10.5281/zenodo.14728851, the processed data and code for analysis are available on https://github.com/craggASAP/Pavlovian_Associations.git.

Research involving human participants, their data, or biological material

Policy information about studies with human participants or human data. See also policy information about sex, gender (identity/presentation), and sexual orientation and race, ethnicity and racism.

Reporting on sex and gender	No human subjects were used
Reporting on race, ethnicity, or other socially relevant groupings	n/a
Population characteristics	n/a
Recruitment	n/a
Ethics oversight	n/a

Note that full information on the approval of the study protocol must also be provided in the manuscript.

Field-specific reporting

Please select the one below that is the best fit for your research. If you are not sure, read the appropriate sections before making your selection.

☒ Life sciences ☐ Behavioural & social sciences ☐ Ecological, evolutionary & environmental sciences

For a reference copy of the document with all sections, see nature.com/documents/nr-reporting-summary-flat.pdf

Life sciences study design

All studies must disclose on these points even when the disclosure is negative.

Sample size	Our sample sizes are consistent with or larger than similar publications using comparable methods (see refs: 37,38, 53, 66)
Data exclusions	Individual fibers were excluded from analysis based on localization outside the striatum or poor signal to noise ratio. Otherwise, no animals were excluded from analysis.
Replication	consistent results were observed across multiple experimental subjects in different experimental cohorts
Randomization	there was no requirement for randomization
Blinding	blinding to experimental condition was not relevant

Reporting for specific materials, systems and methods

We require information from authors about some types of materials, experimental systems and methods used in many studies. Here, indicate whether each material, system or method listed is relevant to your study. If you are not sure if a list item applies to your research, read the appropriate section before selecting a response.

Materials & experimental systems

n/a	Involved in the study
☒	☐ Antibodies
☒	☐ Eukaryotic cell lines
☒	☐ Palaeontology and archaeology
☐	☒ Animals and other organisms
☒	☐ Clinical data
☒	☐ Dual use research of concern
☒	☐ Plants

Methods

n/a	Involved in the study
☒	☐ ChIP-seq
☒	☐ Flow cytometry
☒	☐ MRI-based neuroimaging

Animals and other research organisms

Policy information about studies involving animals; ARRIVE guidelines recommended for reporting animal research, and Sex and Gender in Research

Laboratory animals	the following mice lines were used: Wild Type C57BL6 /J (Jackson Labs, strain #00664); heterozygous ChAT-cre mice (ChAT-IRES-Cre, Jackson Labs, strain# 006410). Ages ranged 3-5 months
Wild animals	no wild animals were used
Reporting on sex	this study used both female and male mice, 60% of the mice used in this study were male
Field-collected samples	N/A
Ethics oversight	All animal care and experimental procedures were performed in accordance with protocols approved by the Boston University Institutional Animal Care and Use Committee (protocol no. 201800554) and in compliance with the Guide for Animal Care and Use of Laboratory Animals.

Note that full information on the approval of the study protocol must also be provided in the manuscript.

Plants

Seed stocks	N/A
Novel plant genotypes	N/A
Authentication	N/A
